# Supplementary material for: Splice-Junction-Based Mapping of Alternative Isoforms in the Human Proteome
Source: Cell Rep. Author manuscript; Available in PMC 2020 Jan 15. (PMC6961840; doi:10.1016/j.celrep.2019.11.026)
Supplement: 2 [file NIHMS1546469-supplement-2.zip › DF1/PXD006675/RightVentricle/RightVentricle_22_MYBPC3_AITQLLCETEGR.pdf]

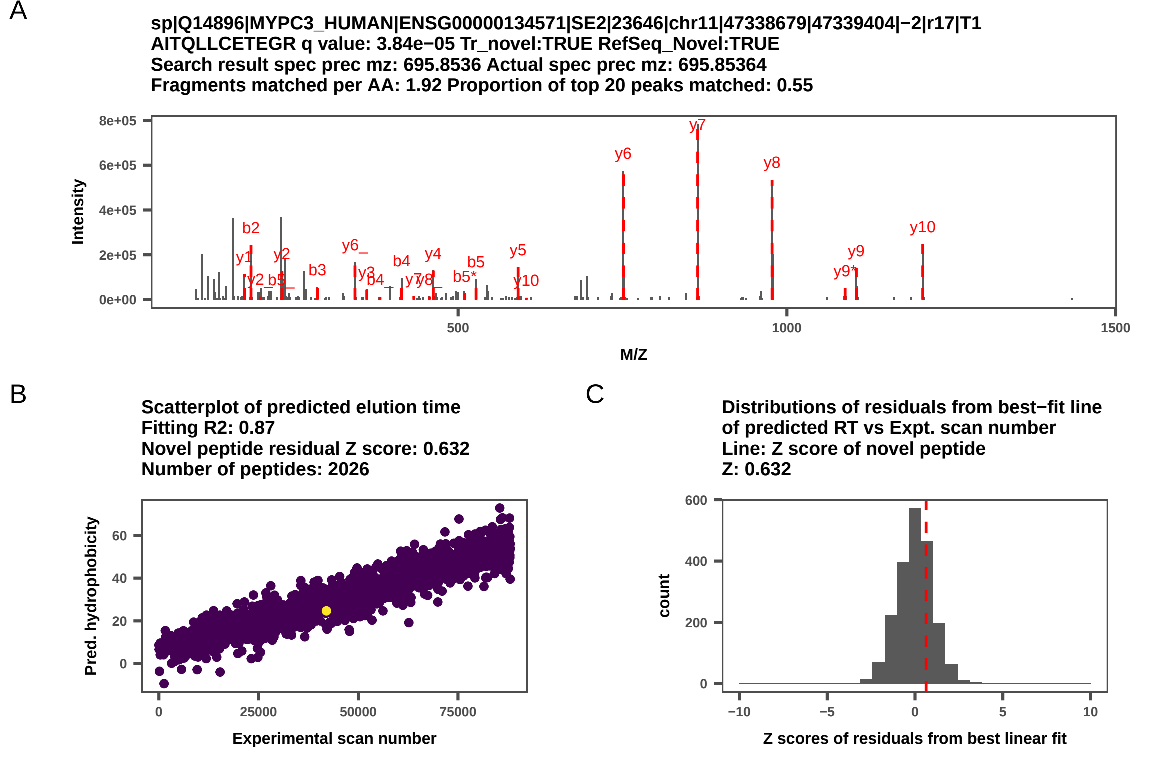
A
sp|Q14896|MYPC3_HUMAN|ENSG00000134571|SE2|23646|chr11|47338679|47339404|−2|r17|T1
AITQLLCETEGR q value: 3.84e−05 Tr_novel:TRUE RefSeq_Novel:TRUE
Search result spec prec mz: 695.8536 Actual spec prec mz: 695.85364
Fragments matched per AA: 1.92 Proportion of top 20 peaks matched: 0.55
Intensity
8e+05
6e+05
4e+05
2e+05
0e+00
M/Z
500
1000
1500
b2
y1
y2
y2_
b5_
b3
y6_
y3
b4_
b4
y7
y8_
y4
b5
b5*
y5
y10
y6
y7
y8
y9
y9*
y10
B
Scatterplot of predicted elution time
Fitting R2: 0.87
Novel peptide residual Z score: 0.632
Number of peptides: 2026
Pred. hydrophobicity
60
40
20
0
Experimental scan number
0
25000
50000
75000
C
Distributions of residuals from best−fit line
of predicted RT vs Expt. scan number
Line: Z score of novel peptide
Z: 0.632
count
600
400
200
0
Z scores of residuals from best linear fit
−10
−5
0
5
10
